# (1*R*,3*S*)-3-(1*H*-Benzo[*d*]imidazol-2-yl)-1,2,2-tri­methyl­cyclo­pentane-1-carb­oxy­lic acid as a new anti-diabetic active pharmaceutical ingredient

**DOI:** 10.1107/S2056989020010439

**Published:** 2020-08-04

**Authors:** Sergiy M. Kovalenko, Irina S. Konovalova, Sergiy I. Merzlikin, Vladimir P. Chuev, Dmitry V. Kravchenko

**Affiliations:** a V.N. Karazin Kharkiv National University, 4 Svobody Sq., Kharkiv, 61077, Ukraine; b State Scientific Institution Institute for Single Crystals of the National Academy of Sciences of Ukraine, 61001, Kharkov, Ukraine; c National University of Pharmacy, 53 Pushkinska St., Kharkiv, 61002, Ukraine; d Federal State Autonomous Educational Institution of Higher Education Belgorod State University, 85, Pobedy St., Belgorod, 308015, Russian Federation; e Experimental Plant for Dental Materials Vladmiva, 81d, Michurin St., Belgorod, 308015, Russian Federation; f Chemical Diversity Research Institute, 2A Rabochaya St., Khimki, Moscow Region, 141400, Russian Federation

**Keywords:** crystal structure, Hirshfeld surface, 1*H*-benzo[*d*]imidazol-2-yl-1,2,2-tri­methyl­cyclo­pentane-carb­oxy­lic acid, active pharmaceutical ingredient, anti-diabetic agents, type 2 diabetes

## Abstract

The chiral title compound, C_16_H_20_N_2_O_2_, which can be used for producing active pharmaceutical ingredients for treatment of type 2 pancreatic diabetes and other pathologies dependent on insulin resistance, was prepared from (1*R*,3*S*)-camphoric acid and *o*-phenyl­enedi­amine. It crystallized from an ethanol solution in the chiral monoclinic *P2_1_* space group. The five-membered ring adopts a twisted conformation with the methyl-substituted C atoms displaced by −0.273 (5) and 0.407 (5) Å from the mean plane through the other three atoms. In the crystal, mol­ecules are linked by O—H⋯N hydrogen bonds, forming chains along the *a*-axis direction. Hirshfeld surface analysis and two-dimensional fingerprint plots were used to analyze the inter­molecular contacts present in the crystal.

## Abstract 2

The chiral title compound, which can be used for producing active pharmaceutical ingredients for treatment of type 2 pancreatic diabetes and other pathologies dependent on insulin resistance, was prepared from (1*R*,3S)-camphoric acid and *o*-phenyl­enedi­amine.

## Chemical context   

The incidence of diabetes has taken on the character of an epidemic in the world. According to the forecasts of the World Health Organization, the number of patients with diabetes will double and reach 300 million people by 2025 (Zimmet *et al.*, 2001 ▸). In this regard, developing and introducing new anti­diabetic drugs is of great importance.

A great number of camphoric acid as well as benzimidazole derivatives exhibit different types of biological activities (Merzlikin *et al.*, 2008 ▸; Ivachtchenko *et al.*, 2002 ▸, 2019 ▸; Kovalenko *et al.*, 1998 ▸).
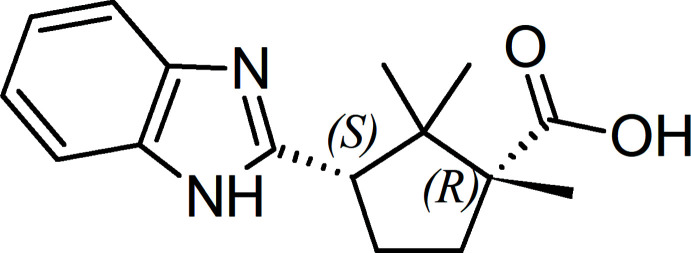


Our research on the mol­ecular design, construction and synthesis of new benzimidazole derivatives of 1,2,2,3-tetra­methyl­cyclo­pentane-1-carb­oxy­lic acid has shown that (1*R*,3*S*)-3-(1*H*-benzo[*d*]imidazol-2-yl)-1,2,2-tri­methyl­cyclo­pentane-1-carb­oxy­lic acid, **4**, exhibits pronounced anti­diabetic activity and, in particular, anti­hyperglycemic effect, which reduces insulin resistance and restores the physiological function of pancreatic β-cells (Jain *et al.*, 2009 ▸; Chuev *et al.*, 2017 ▸).

Racemic and enanti­omeric crystals are known to possess different activities, which is very important in the pharmaceutical industry. We have found that the disadvantage of the (±) and (-) forms of compound **4** described in the patent of Merzlikin *et al.* (2009 ▸) is their poor bioavailability as compared to the (+) form. To obtain (1*R*,3*S*)-3-(1*H*-benzo[*d*] imidazol-2-yl)-1,2,2-tri­methyl­cyclo­pentane-1-carb­oxy­lic acid **4**, the enanti­omerically pure (1*R*,3*S*)-camphoric acid **1** was used.

**Figure 1:**
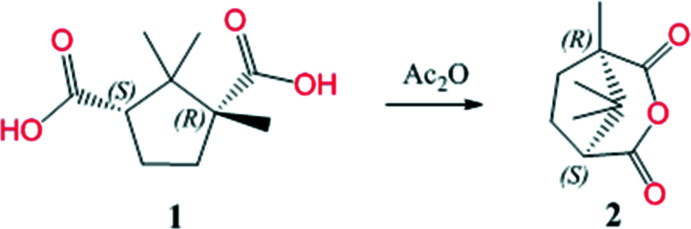
Synthesis of (1*R*,5*S*)-1,8,8-trimethyl-3-oxabi­cyclo­[3.2.1]octane-2,4-dione, **2**.

In the first stage, (1*R*,5*S*)-1,8,8-trimethyl-3-oxabi­cyclo­[3.2.1]octane-2,4-dione [d-(+)-camphoric anhydride] **2** was obtained by refluxing a mixture of (1*R*,3*S*)-camphoric acid and acetic anhydride for 2 h (Dong *et al.*, 2016 ▸) (Fig. 1 ▸).

**Figure 2:**
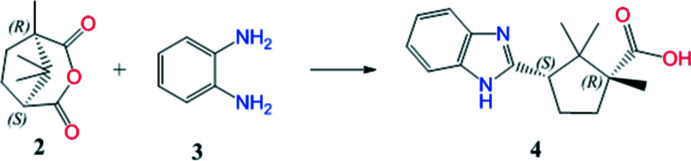
Synthesis of (1*R*,3*S*)-3-(1*H*-benzo[*d*]imidazol-2-yl)-1,2,2- tri­methyl­cyclo­pentane-1-carb­oxy­lic acid, **4**.

In the second stage, the synthesis of (1*R*,3*S*)-3-(1*H*-benzo[d]imidazol-2-yl)-1,2,2- tri­methyl­cyclo­pentane-1-carb­oxy­lic acid **4** was carried out according to Fig. 2 ▸ *via* cyclo­condensation of d-(+)-camphoric anhydride **2** with *o*-phenyl­enedi­amine **3** in a mixture of toluene and DMF (383 K) by refluxing for several hours (Fig. 2 ▸).

It should be noted that during the synthesis, the configuration of the chiral centers did not change and the structure of the title mol­ecule was unambiguously confirmed by X-ray analysis.

## Structural commentary   

**Figure 3:**
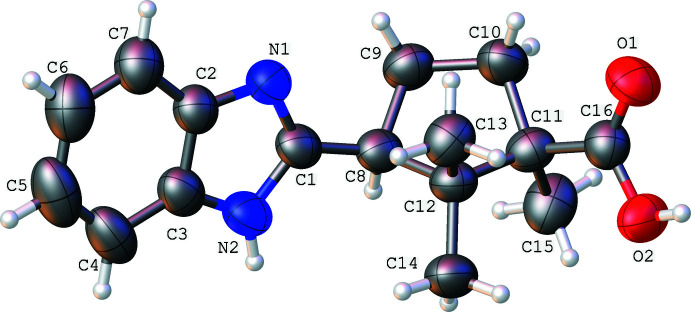
The mol­ecular structure of the title compound **4** with the atom labeling. Displacement ellipsoids are drawn at the 50% probability level.

The asymmetric unit contains one mol­ecule of the title compound **4** (Fig. 3 ▸). The bicyclic fragment is planar with a maximum deviation of 0.016 (6) Å (for atom C16). The saturated five-membered ring adopts a twisted conformation in which the deviations of atoms C11 and C12 from the mean-square plane through the remaining ring atoms are −0.273 (5) and 0.407 (5) Å, respectively. The cyclo­pentane ring is turned in relation to the N1—C1 endocyclic bond, the N1—C1—C8—C9 torsion angle being −30.0 (7)°. It can be assumed that the weak intra­molecular C9—H9*B*⋯N1 (H⋯N = 2.53 Å, C—H⋯N = 108°) hydrogen bond additionally stabilizes such a location of the saturated ring. The methyl group on the C11 atom is located in the axial position [C9—C10—C11—C15 = 87.5 (6)°]. The carboxyl group has an equatorial orientation and is almost coplanar to the endocyclic C10–C11 bond [the C9—C10—C11—C16 and C10—C11—C16—O1 torsion angles are −150.8 (5) and 13.9 (8)°, respectively]. This position is stabilized by the formation of weak intra­molecular C10—H10*A*⋯O1 and C15—H15*B*⋯O2 hydrogen bonds between the vicinal and geminal substituents (H⋯O = 2.43 and 2.42 Å, C—H⋯O = 103 and 100°, respectively). The presence of geminal substituents on neighboring atoms of the pentane ring leads to significant steric repulsion (the shortened intra­molecular contacts are given in Table 1 ▸), which causes elongation of the C8—C12 bond to 1.571 (7) Å, compared with its mean value of 1.556 Å (Burgi *et al.*, 1994 ▸).

## Supra­molecular features   

**Figure 4:**
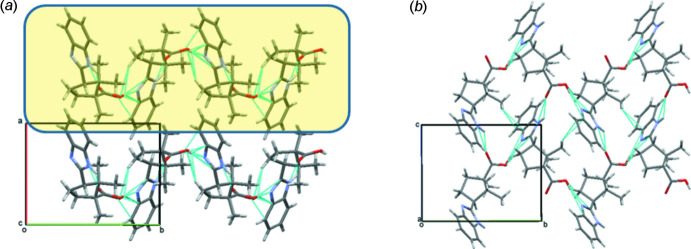
(*a*) View of the structure of compound **4** down the *b* axis and (*b*) hydrogen bonds within a layer in the crystal of **4**.

In the crystal, mol­ecules of **4** form layers parallel to the (100) plane as a result of the strong N2—H⋯O1 and O2—H⋯N1 and weak C14—H14*C*⋯C2(π) inter­molecular hydrogen bonds (Table 2 ▸, Fig. 4 ▸ *a*,b). The neighboring layers are not bound any specific inter­actions (Fig. 4 ▸ *a*). It is inter­esting to note that the mol­ecules are linked by hydrogen bonds that use the O—H⋯N heterosynthon instead of the carb­oxy­lic acid dimer homosynthon. Despite the presence of an aromatic ring in the mol­ecule, no stacking inter­actions are observed in the crystal of **4**. Instead of π– π inter­actions, C—H⋯π inter­actions are formed (Table 2 ▸, Fig. 4 ▸ *b*).

## Hirshfeld surface analysis   

**Figure 5:**
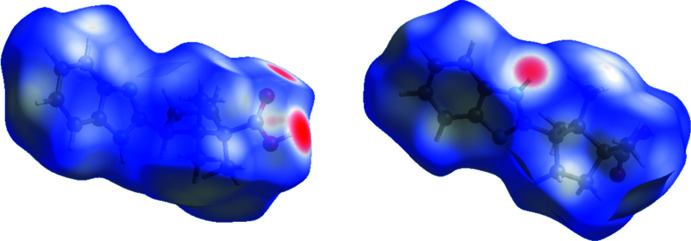
Two orientations of the Hirshfeld surface for the title compound mapped over *d*_norm_.

*Crystal Explorer* 17.5 (Turner *et al.*, 2017 ▸) was used to analyze the inter­actions in the crystal and fingerprint plots mapped over *d*_norm_ (Figs. 5 ▸ and 6 ▸) were generated. The mol­ecular Hirshfeld surfaces were obtained using a standard (high) surface resolution with the three-dimensional *d*_norm_ surfaces mapped over a fixed color scale of −0.716 (red) to 1.406 (blue) a.u. The red spots indicate regions of donor–acceptor inter­actions or short contacts. There are three red spots in the *d*_norm_ surface for **4** (Fig. 5 ▸), which correspond to the inter­actions listed in Table 2 ▸.

**Figure 6:**
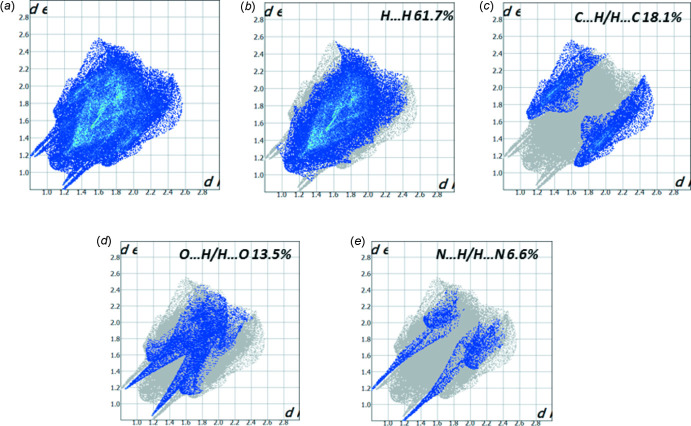
(*a*) The two-dimensional fingerprint plot for compound **4**, and those delineated into (*b*) H⋯H (61.7%), (*c*) C⋯H/H⋯C (18.1%), (*d*) O⋯H/H⋯O (13.5%) and (*e*) N⋯H/H⋯N (6.6%) contacts.

All of the inter­molecular inter­actions of the title compound are shown in the two-dimensional fingerprint plot presented in Fig. 6 ▸ *a*. The fingerprint plots indicate that the principal contributions are from H⋯H (61.7%; Fig. 6 ▸ *b*), C⋯H/H⋯C (18.1%; Fig. 6 ▸ *c*), O⋯H/H⋯O (13.5%; Fig. 6 ▸ *d*) and N⋯H/H⋯N (6.6%; Fig. 6 ▸ *e*) contacts. The H⋯H inter­actions appear in the middle of the plot scattered over a large area, while the C⋯H/H⋯C contacts are represented by the ‘wings’ of the plot. O⋯H/H⋯O inter­actions appear as inner spikes and the N⋯H/H⋯N contacts, corresponding to the O—H⋯N inter­action, are represented by a pair of sharp outer spikes, which indicate they are the strongest inter­actions in the crystal of **4**.

## Database survey   

A search of the Cambridge Structural Database (CSD, Version 5.41, November 2019; Groom *et al.*, 2016 ▸) for the 1,2,2-tri­methyl­cyclo­pentane-1-carb­oxy­lic acid skeleton yielded 27 hits. Only one structure involves a benzo­thia­zol-2′-yl ring in position 3, *viz.* (1*R*,3*S*)-(+)-*cis*-1,3-bis­(benzo­thia­zol-2′-yl)-1,2,2-tri­methyl­cyclo­pentane (CSD refcode XUMXIM; Gilbert *et al.*, 2002 ▸). The cyclo­pentane ring has the twist conformation with the atoms C1 and C4 displaced by 0.48 (1) and −0.26 (2) Å from the mean plane through the other three atoms [*cf.* 0.407 (5) Å and −0.273 (5) Å in the title compound].

## Synthesis and crystallization   

**(1** ***R*** **,3** ***S*** **)-3-(1** ***H*** **-Benzo[** ***d*** **]imidazol-2-yl)-1,2,2-tri­methyl­cyclo­pentane-1-carb­oxy­lic acid**, **4**

In a glass reactor equipped with a Dean–Stark receiver, d-(+)-camphoric anhydride **2** (2.20 kg, 12.1 mol), *o*-phenyl­enedi­amine **3** (1.31 kg, 12.1 mol), toluene (11.46 L) and di­methyl­formamide (0.91 L) were charged. Under stirring, the reaction mixture was heated to boiling (383 K). The mixture was refluxed and the released water was collected in the Dean–Stark receiver. When the removal of water had finished, the reaction mixture was cooled to room temperature. The precipitate that formed was filtered *in vacuo* using a Nutsche filter. The precipitate was thoroughly squeezed, washed twice with toluene (1.4 L) and re-squeezed. Then the precipitate was washed on the filter with 70% water–ethanol (3.7 L), heated to a temperature of 348±5 K. Finally, the precipitate of the product **4** was thoroughly squeezed and dried at 343 K for 4 h, yielding 2.41 kg (73.2%) of a white crystal-like powder that is practically insoluble in water, soluble in 96% alcohol, m.p. 527–528 K. UV (ethanol) λmax (∊): 204 nm (48960), 245 nm (6800), 275 nm (9160), 281 nm (9320); IR (KBr): ν (cm^−1^) 3450 (O—H), 3286 (N—H), 2970, 2935, 2887 (C—H), 1673 (C=O), 1529, 1456, 1436, 1373, 1279, 1358, 1167, 1124, 1057, 740; ^1^H NMR (400 MHz, DMSO-*d*_6_) δ 12.22 (*s.br*, 1H, OH), 12.10 (*s.br*, 1H, NH), 7.52 (*s.br*, 1H, H-4, H-7), 7.44 (*s.br*, 1H, H-4, H-7), 7.10 (*t*, *J* = 4.5 Hz, 2H, H-5, H-6), 3.41–3.31 (*m*, 1H, CH), 2.64–2.54 (*m*, 1H,CH), 2.43–2.33 (*m*, 1H, CH), 2.05–1.95 (*m*, 1H, CH), 1.55–1.45 (*m*, 1H, CH), 1.25 (*s*, 3H, CH3), 1.14 (*s*, 3H, CH_3_), 0.61 (*s*, 3H, CH_3_); LC/MS *m*/*z* (%): 273.2 [*M*H]+ (100); found, %: C 70.88; H 7.83; N 10.55. C_16_H_20_N_2_O_2_. Calculated, %: C 70.56; H 7.40; N 10.29.

**Figure 7:**
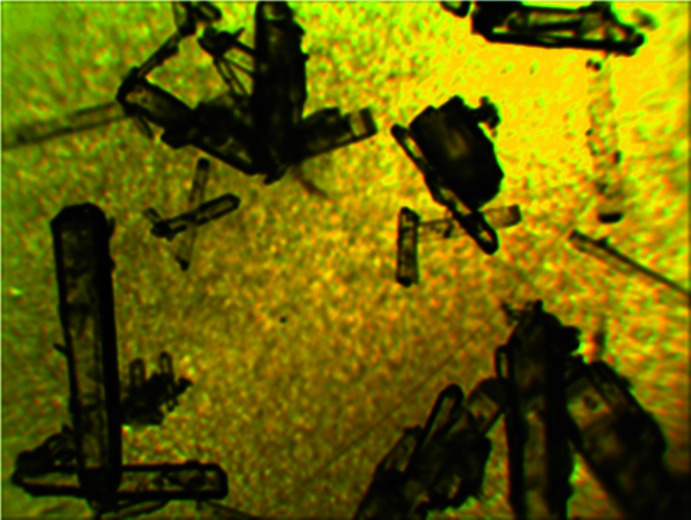
Crystals of the title compound **4**.

Further crystallization by slow evaporation of an ethanol solution was carried out to provide single block-like colorless crystals (Fig. 7 ▸) suitable for X-ray diffraction analysis.

## Refinement   

Crystal data, data collection and structure refinement details are summarized in Table 3 ▸. H atoms were included in calculated positions and treated as riding on their parent C atom: C—H = 0.82–0.98 Å with *U*_iso_(H) = 1.5*U*_eq_(C-methyl and O-hydrox­yl) and 1.2*U*_eq_(C) for other H atoms. The Flack parameter cannot be determined reliably, because there is no X-ray anomalous scattering because of the absence of heavy atoms in the mol­ecule.

## Supplementary Material

Crystal structure: contains datablock(s) I. DOI: 10.1107/S2056989020010439/dj2007sup1.cif

Structure factors: contains datablock(s) I. DOI: 10.1107/S2056989020010439/dj2007Isup2.hkl

Supporting information file. DOI: 10.1107/S2056989020010439/dj2007Isup3.cml

CCDC reference: 2019647

Additional supporting information:  crystallographic information; 3D view; checkCIF report

## Figures and Tables

**Table 1:** Intra­molecular short contacts (Å) in compound **4** together with the sums of the respective van der Waals radii The van der Waals radii sum values (Zefirov *et al.*, 1997 ▸) are given in parentheses.

H8⋯H15*A*	2.26 (2.34)	H15*B*⋯H14*C*	2.32 (2.34)
H13*C*⋯C1	2.57 (2.87)	H13*B*⋯C16	2.54 (2.87)

**Table 2:** Hydrogen-bond geometry (Å, °)

*D*—H⋯*A*	*D*—H	H⋯*A*	*D*⋯*A*	*D*—H⋯*A*
O2—H2⋯N1^i^	0.82	1.82	2.631 (6)	170
N2—H2*A*⋯O1^ii^	0.86	2.16	2.871 (6)	140
C14—H14*C*⋯C2^iii^	0.96	2.81	3.439 (8)	124

Symmetry codes: (i) 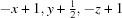; (ii) 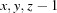; (iii) 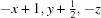.

**Table 3:** Experimental details

Crystal data	
Chemical formula	C_16_H_20_N_2_O_2_
*M*_r_	272.34
Crystal system, space group	Monoclinic, *P*2_1_
Temperature (K)	293
*a*, *b*, *c* (Å)	7.9805 (7), 10.8671 (8), 8.4912 (7)
β (°)	94.056 (7)
*V* (Å^3^)	734.55 (10)
*Z*	2
Radiation type	Mo *K*α
μ (mm^−1^)	0.08
Crystal size (mm)	0.3 × 0.2 × 0.1

Data collection	
Diffractometer	Rigaku Oxford Diffraction Xcalibur, Sapphire3
Absorption correction	Multi-scan (*CrysAlis PRO*; Rigaku OD, 2018 ▸)
*T*_min_, *T*_max_	0.182, 1.000
No. of measured, independent and observed [*I* > 2σ(*I*)] reflections	4859, 2455, 1731
*R*_int_	0.058
(sin θ/λ)_max_ (Å^−1^)	0.594

Refinement	
*R*[*F* ^2^ > 2σ(*F* ^2^)], *wR*(*F* ^2^), *S*	0.065, 0.180, 1.04
No. of reflections	2455
No. of parameters	185
No. of restraints	1
H-atom treatment	H-atom parameters constrained
Δρ_max_, Δρ_min_ (e Å^−3^)	0.15, −0.19
Absolute structure	Flack *x* determined using 459 quotients [(*I* ^+^)−(*I* ^−^)]/[(*I* ^+^)+(*I* ^−^)] (Parsons et al., 2013 ▸)
Absolute structure parameter	−1.8 (10)

Computer programs: *CrysAlis PRO* (Rigaku OD, 2018 ▸), *SHELXT* (Sheldrick, 2015*a* ▸), *SHELXL* (Sheldrick, 2015*b* ▸), *OLEX2* (Dolomanov *et al.*, 2009 ▸).

## supplementary crystallographic information

### Crystal data

**Table:**

C_16_H_20_N_2_O_2_	*F*(000) = 292
*M**_r_* = 272.34	*D*_x_ = 1.231 Mg m^−^^3^
Monoclinic, *P*2_1_	Mo *K*α radiation, λ = 0.71073 Å
*a* = 7.9805 (7) Å	Cell parameters from 748 reflections
*b* = 10.8671 (8) Å	θ = 3.6–20.3°
*c* = 8.4912 (7) Å	µ = 0.08 mm^−^^1^
β = 94.056 (7)°	*T* = 293 K
*V* = 734.55 (10) Å^3^	Plate, colourless
*Z* = 2	0.3 × 0.2 × 0.1 mm

### Data collection

**Table:**

Rigaku Oxford Diffraction Xcalibur, Sapphire3 diffractometer	2455 independent reflections
Radiation source: fine-focus sealed X-ray tube, Enhance (Mo) X-ray Source	1731 reflections with *I* > 2σ(*I*)
Graphite monochromator	*R*_int_ = 0.058
Detector resolution: 16.1827 pixels mm^-1^	θ_max_ = 25.0°, θ_min_ = 3.1°
ω scans	*h* = −9→8
Absorption correction: multi-scan (CrysAlisPro; Rigaku OD, 2018)	*k* = −12→11
*T*_min_ = 0.182, *T*_max_ = 1.000	*l* = −9→10
4859 measured reflections	

### Refinement

**Table:**

Refinement on *F*^2^	Hydrogen site location: inferred from neighbouring sites
Least-squares matrix: full	H-atom parameters constrained
*R*[*F*^2^ > 2σ(*F*^2^)] = 0.065	*w* = 1/[σ^2^(*F*_o_^2^) + (0.0794*P*)^2^] where *P* = (*F*_o_^2^ + 2*F*_c_^2^)/3
*wR*(*F*^2^) = 0.180	(Δ/σ)_max_ < 0.001
*S* = 1.04	Δρ_max_ = 0.15 e Å^−^^3^
2455 reflections	Δρ_min_ = −0.19 e Å^−^^3^
185 parameters	Absolute structure: Flack *x* determined using 459 quotients [(*I*^+^)-(*I*^-^)]/[(*I*^+^)+(*I*^-^)] (Parsons et al., 2013)
1 restraint	Absolute structure parameter: −1.8 (10)
Primary atom site location: dual	

### Special details

**Table:**

Geometry. All esds (except the esd in the dihedral angle between two l.s. planes) are estimated using the full covariance matrix. The cell esds are taken into account individually in the estimation of esds in distances, angles and torsion angles; correlations between esds in cell parameters are only used when they are defined by crystal symmetry. An approximate (isotropic) treatment of cell esds is used for estimating esds involving l.s. planes.

### Fractional atomic coordinates and isotropic or equivalent isotropic displacement parameters (Å^2^)

**Table:**

	*x*	*y*	*z*	*U*_iso_*/*U*_eq_	
O1	0.3639 (6)	0.5406 (4)	0.7106 (5)	0.0801 (12)	
O2	0.2632 (6)	0.7051 (3)	0.5806 (5)	0.0755 (12)	
H2	0.289977	0.738749	0.665107	0.113*	
N1	0.6692 (5)	0.3376 (4)	0.1635 (5)	0.0593 (11)	
N2	0.5767 (6)	0.4717 (4)	−0.0168 (5)	0.0673 (13)	
H2A	0.510294	0.522765	−0.067258	0.081*	
C1	0.5454 (7)	0.4112 (5)	0.1190 (6)	0.0584 (13)	
C2	0.7894 (7)	0.3527 (5)	0.0550 (7)	0.0604 (14)	
C3	0.7336 (8)	0.4365 (5)	−0.0588 (7)	0.0654 (14)	
C4	0.8269 (10)	0.4678 (6)	−0.1848 (8)	0.0831 (19)	
H4	0.786965	0.523797	−0.261269	0.100*	
C5	0.9807 (11)	0.4124 (8)	−0.1914 (9)	0.096 (2)	
H5	1.047951	0.432369	−0.272757	0.115*	
C6	1.0379 (9)	0.3261 (7)	−0.0773 (9)	0.090 (2)	
H6	1.142413	0.289532	−0.084821	0.108*	
C7	0.9429 (8)	0.2942 (7)	0.0460 (8)	0.0759 (17)	
H7	0.980342	0.235604	0.120267	0.091*	
C8	0.3928 (7)	0.4359 (5)	0.2036 (6)	0.0580 (13)	
H8	0.300846	0.455808	0.125078	0.070*	
C9	0.3372 (7)	0.3271 (5)	0.3025 (7)	0.0679 (15)	
H9A	0.245558	0.283181	0.246369	0.082*	
H9B	0.429868	0.270412	0.324145	0.082*	
C10	0.2806 (9)	0.3803 (5)	0.4556 (8)	0.0716 (16)	
H10A	0.360495	0.359711	0.543071	0.086*	
H10B	0.171604	0.347548	0.477382	0.086*	
C11	0.2707 (6)	0.5205 (5)	0.4334 (7)	0.0597 (14)	
C12	0.4144 (6)	0.5465 (5)	0.3225 (6)	0.0575 (12)	
C13	0.5865 (7)	0.5408 (6)	0.4150 (7)	0.0707 (15)	
H13A	0.597970	0.463626	0.469595	0.106*	
H13B	0.595652	0.606974	0.490044	0.106*	
H13C	0.673547	0.548409	0.343119	0.106*	
C14	0.3983 (9)	0.6692 (5)	0.2366 (8)	0.0764 (17)	
H14A	0.491174	0.679383	0.171902	0.115*	
H14B	0.398423	0.734744	0.312382	0.115*	
H14C	0.295039	0.670837	0.171336	0.115*	
C15	0.0964 (7)	0.5560 (7)	0.3588 (8)	0.0811 (18)	
H15A	0.079109	0.518462	0.256632	0.122*	
H15B	0.089416	0.643848	0.348102	0.122*	
H15C	0.011624	0.527941	0.425166	0.122*	
C16	0.3042 (6)	0.5877 (5)	0.5895 (7)	0.0612 (15)	

### Atomic displacement parameters (Å^2^)

**Table:**

	*U*^11^	*U*^22^	*U*^33^	*U*^12^	*U*^13^	*U*^23^
O1	0.100 (3)	0.086 (3)	0.053 (2)	0.005 (2)	−0.011 (2)	0.006 (2)
O2	0.103 (3)	0.063 (2)	0.058 (3)	0.012 (2)	−0.014 (2)	−0.0111 (18)
N1	0.067 (3)	0.060 (3)	0.051 (3)	0.004 (2)	0.001 (2)	0.001 (2)
N2	0.083 (3)	0.067 (3)	0.050 (3)	0.003 (2)	−0.004 (2)	0.006 (2)
C1	0.070 (3)	0.060 (3)	0.043 (3)	−0.001 (3)	−0.006 (2)	−0.001 (2)
C2	0.063 (3)	0.066 (3)	0.051 (3)	−0.002 (3)	0.000 (2)	−0.006 (3)
C3	0.078 (4)	0.063 (3)	0.055 (3)	−0.010 (3)	0.004 (3)	−0.005 (3)
C4	0.104 (5)	0.088 (4)	0.059 (4)	−0.026 (4)	0.016 (3)	−0.001 (3)
C5	0.098 (5)	0.119 (6)	0.073 (5)	−0.039 (5)	0.027 (4)	−0.019 (4)
C6	0.075 (4)	0.113 (6)	0.083 (5)	−0.011 (4)	0.012 (4)	−0.026 (5)
C7	0.071 (4)	0.090 (4)	0.066 (4)	0.000 (3)	0.001 (3)	−0.011 (3)
C8	0.068 (3)	0.056 (3)	0.049 (3)	0.002 (2)	−0.007 (2)	−0.002 (2)
C9	0.074 (4)	0.055 (3)	0.074 (4)	−0.001 (3)	0.003 (3)	−0.001 (3)
C10	0.088 (4)	0.061 (3)	0.066 (4)	−0.005 (3)	0.008 (3)	0.005 (3)
C11	0.056 (3)	0.055 (3)	0.067 (4)	0.003 (2)	−0.004 (2)	0.000 (3)
C12	0.061 (3)	0.052 (3)	0.058 (3)	0.001 (2)	−0.002 (2)	0.003 (2)
C13	0.064 (3)	0.071 (3)	0.075 (4)	0.000 (3)	−0.005 (3)	−0.010 (3)
C14	0.102 (5)	0.058 (3)	0.069 (4)	0.007 (3)	0.003 (3)	0.007 (3)
C15	0.063 (3)	0.097 (4)	0.081 (4)	0.005 (4)	−0.012 (3)	−0.019 (4)
C16	0.057 (3)	0.068 (4)	0.058 (4)	0.001 (3)	0.001 (3)	−0.005 (3)

### Geometric parameters (Å, º)

**Table:**

O1—C16	1.216 (6)	C5—C6	1.401 (11)
O2—C16	1.319 (6)	C6—C7	1.380 (9)
N1—C1	1.307 (7)	C8—C9	1.533 (8)
N1—C2	1.386 (7)	C8—C12	1.571 (7)
N2—C1	1.366 (7)	C9—C10	1.520 (9)
N2—C3	1.381 (8)	C10—C11	1.536 (8)
C1—C8	1.481 (8)	C11—C12	1.560 (8)
C2—C3	1.378 (8)	C11—C15	1.537 (7)
C2—C7	1.387 (8)	C11—C16	1.520 (8)
C3—C4	1.388 (8)	C12—C13	1.534 (7)
C4—C5	1.372 (10)	C12—C14	1.521 (8)
			
C1—N1—C2	106.3 (5)	C10—C9—C8	106.8 (5)
C1—N2—C3	107.9 (5)	C9—C10—C11	106.7 (5)
N1—C1—N2	111.0 (5)	C10—C11—C12	102.7 (4)
N1—C1—C8	127.0 (5)	C10—C11—C15	109.7 (5)
N2—C1—C8	121.9 (5)	C15—C11—C12	112.9 (5)
N1—C2—C7	129.6 (6)	C16—C11—C10	111.4 (5)
C3—C2—N1	109.9 (5)	C16—C11—C12	110.4 (4)
C3—C2—C7	120.5 (6)	C16—C11—C15	109.7 (4)
N2—C3—C4	132.6 (6)	C11—C12—C8	101.4 (4)
C2—C3—N2	104.9 (5)	C13—C12—C8	110.6 (4)
C2—C3—C4	122.5 (6)	C13—C12—C11	110.7 (4)
C5—C4—C3	117.0 (7)	C14—C12—C8	111.1 (4)
C4—C5—C6	120.9 (6)	C14—C12—C11	114.0 (5)
C7—C6—C5	121.6 (7)	C14—C12—C13	108.8 (5)
C6—C7—C2	117.5 (7)	O1—C16—O2	122.4 (5)
C1—C8—C9	113.9 (5)	O1—C16—C11	124.8 (5)
C1—C8—C12	113.2 (4)	O2—C16—C11	112.8 (5)
C9—C8—C12	105.1 (4)		
			
N1—C1—C8—C9	−30.0 (7)	C7—C2—C3—N2	177.5 (5)
N1—C1—C8—C12	90.0 (6)	C7—C2—C3—C4	−1.0 (8)
N1—C2—C3—N2	−0.1 (6)	C8—C9—C10—C11	10.7 (7)
N1—C2—C3—C4	−178.6 (5)	C9—C8—C12—C11	−35.1 (5)
N1—C2—C7—C6	179.1 (5)	C9—C8—C12—C13	82.4 (6)
N2—C1—C8—C9	153.8 (5)	C9—C8—C12—C14	−156.6 (5)
N2—C1—C8—C12	−86.2 (6)	C9—C10—C11—C12	−32.7 (6)
N2—C3—C4—C5	−178.8 (6)	C9—C10—C11—C15	87.5 (6)
C1—N1—C2—C3	−1.0 (6)	C9—C10—C11—C16	−150.8 (5)
C1—N1—C2—C7	−178.3 (6)	C10—C11—C12—C8	41.1 (5)
C1—N2—C3—C2	1.1 (6)	C10—C11—C12—C13	−76.3 (5)
C1—N2—C3—C4	179.4 (6)	C10—C11—C12—C14	160.6 (5)
C1—C8—C9—C10	140.0 (5)	C10—C11—C16—O1	13.9 (8)
C1—C8—C12—C11	−160.0 (4)	C10—C11—C16—O2	−166.9 (5)
C1—C8—C12—C13	−42.5 (6)	C12—C8—C9—C10	15.6 (6)
C1—C8—C12—C14	78.5 (6)	C12—C11—C16—O1	−99.5 (6)
C2—N1—C1—N2	1.7 (6)	C12—C11—C16—O2	79.7 (5)
C2—N1—C1—C8	−174.8 (5)	C15—C11—C12—C8	−76.9 (5)
C2—C3—C4—C5	−0.8 (9)	C15—C11—C12—C13	165.7 (5)
C3—N2—C1—N1	−1.8 (6)	C15—C11—C12—C14	42.6 (6)
C3—N2—C1—C8	174.9 (5)	C15—C11—C16—O1	135.5 (6)
C3—C2—C7—C6	2.0 (8)	C15—C11—C16—O2	−45.3 (7)
C3—C4—C5—C6	1.4 (10)	C16—C11—C12—C8	160.0 (4)
C4—C5—C6—C7	−0.4 (10)	C16—C11—C12—C13	42.6 (6)
C5—C6—C7—C2	−1.3 (9)	C16—C11—C12—C14	−80.5 (5)

### Hydrogen-bond geometry (Å, º)

**Table:**

*D*—H···*A*	*D*—H	H···*A*	*D*···*A*	*D*—H···*A*
O2—H2···N1^i^	0.82	1.82	2.631 (6)	170
N2—H2*A*···O1^ii^	0.86	2.16	2.871 (6)	140
C14—H14*C*···C2^iii^	0.96	2.81	3.439 (8)	124

Symmetry codes: (i) −*x*+1, *y*+1/2, −*z*+1; (ii) *x*, *y*, *z*−1; (iii) −*x*+1, *y*+1/2, −*z*.
